# The Effect of Lavender Aroma on Anxiety of Patients Having Bone Marrow Biopsy

**DOI:** 10.31557/APJCP.2020.21.3.771

**Published:** 2020-03

**Authors:** Reyhaneh Abbaszadeh, Fariba Tabari, Atefeh Asadpour

**Affiliations:** *School of Nursing and Midwifery, Tehran University of Medical Sciences, Tehran, Iran. *

**Keywords:** Bone marrow biopsy, Anxiety, Aromatherapy, Lavender

## Abstract

**Introduction:**

Bone marrow biopsy is a common procedure for the diagnosis and treatment of hematologic diseases and tumors, which are associated with anxiety. The purpose of this study was to examine the effect of lavender aroma on anxiety of patients having bone marrow biopsy.

**Materials and Methods:**

This study was performed on 80 patients referred to Vali-e-Asr Hospital for bone marrow biopsy. Samples were selected by convenience method and were assigned into intervention and control groups using randomized blocks of 4. Random sequence was generated by RAS software. Several drops of distilled water on a cotton ball was used in the control group and same amount of lavender essential oil on a cotton ball was used in the intervention group. Then, participants in both groups were asked to smell the cotton ball for 15 minutes and then, their anxiety level was measured immediately. The results were analyzed by SPSS software version 25 using covariance analysis and rank regression.

**Results:**

The results showed that, the mean scores of anxiety in the control and intervention groups were 6.3 ± 1.92 and 3.75 ± 1.05, respectively. There was a significant difference (p <0.05) between the two groups in terms of anxiety score.The results showed that there was a significant difference in anxiety score between two groups in terms of variables such as age, gender, physician experience, biopsy history and biopsy site (P <0.05). The results also showed no significant difference between the (p >0.05).

**Conclusion:**

The results of this study showed that bone marrow biopsy is associated with anxiety, and smelling of lavender aroma is effective in reducing anxiety in patients undergoing this procedure. This fragrance can be used by treatment team in hematology and oncology clinics to reduce anxiety caused by bone marrow biopsy.

## Introduction

Bone marrow biopsy is an invasive procedure to determine the characteristics of bone marrow cells, evaluate solid tissue involvement in tumors, and identify hematologic malignancies (Özdemir et al., 2019). This procedure is commonly used in clinics to diagnose and treat hematologic diseases (Park et al., 2007), determine the efficacy of treatment for hematologic disorders, and monitor the recovery process in patients undergoing bone marrow transplantation and chemotherapy (Berenson et al., 2011). Bone marrow biopsy was developed as a procedure in early 19th century and allowed physicians to diagnose bone marrow disorders (Hjortholm et al., 2013). Although this procedure is performed at outpatient clinics (Park et al., 2007, Zahid, 2015), it is painful and causes anxiety in patients (Shabanloei et al., 2010). About 56-70% of patients undergoing this procedure report it as a painful procedure (Yayla and Ozdemir, 2019), with 50-70% of them reporting moderate to severe pain (Rizi et al., 2017). Studies show that 60-80% of patients undergoing this procedure or other surgeries experience high level of anxiety (Kaydu and Gokcek, 2019). Hematologic patients also experience anxiety during the process of diagnosis for various reasons (Karacan et al., 2017). Anxiety is an unpleasant and common emotional experience that individuals may experience, especially when they are hospitalized and exposed to surgical and invasive procedures (Sri et al., 2018). This unpleasant experience may lead to sympathetic stimulation and a change in vital signs, including elevated blood pressure and tachycardia. Anxiety management is essential to control vital symptoms during medical procedures, as it can elevate the severity of pain in medical procedures (Perrot et al., 2011). The anxiety experienced during bone marrow biopsy can increase the experience of pain in patients (Özdemir et al., 2019). 

Anxiety is managed in both pharmacological and non-pharmacological ways. Drug therapy is associated with side effects such as dizziness, fatigue and discomfort, and most patients have to be monitored, which is time-consuming. Therefore, non- pharmacological anxiety management methods like aromatherapy are preferred nowadays (Rizi et al., 2017). Aromatherapy refers to the therapeutic use of aroma of herbal oils (Yayla and Ozdemir, 2019), which is an important part of complementary medicine (Beyliklioğlu and Arslan, 2019). It is also cheap and uncomplicated. This method can be used to reduce anxiety and pain (Yayla and Ozdemir, 2019). Aromatherapy has been widely used in cancer and palliative care to improve quality of life and reduce psychological distress (Hu et al., 2010). Today, nurses in USA use this treatment as part of holistic nursing and in UK, this treatment has been accepted in clinical nursing (Shahnazi et al., 2012).

Lavender oil is one of the commonly used oils in aromatherapy to reduce anxiety and pain. Low toxicity and side effects of lavender have made it a favorite treatment in complementary medicine (Sri et al., 2018). Lavender plant can be used orally, topically and via smelling. The essential oil of lavender is 100 times more effective than the plant itself (Karadag et al., 2017). Lavender is used in medicine as a sedative, narcotic, anti-inflammatory and antidepressant (Khalil et al., 2019). Linalool and Linalool Acetate in lavender stimulate the parasympathetic system, causing sedative and narcotic effects (Karadag et al., 2017). Lavender aroma also stimulates the alpha waves in the brain (Afriani and Rahmawati, 2019). Studies have shown that the effect of lavender oil as a cream or aroma on anxiety is similar to the effects of anti-anxiety drugs. Chlorodiazepoxide has been compared with lavender oil and is been found that both of them have similar effects on anxiety. In another study, the effect of lavender oil on anxiety was compared with lorazepam, and it was found that lavender oil was more effective than lorazepam (Fernandez et al., 2018). Studies have shown the positive effect of lavender oil on anxiety caused by childbirth (Mirzaei et al., 2015, Tafazoli et al., 2011), surgery (Braden et al., 2009), dentistry (Lehrner et al., 2005, Zabirunnisa et al., 2014), breast surgery (Beyliklioğlu and Arslan, 2019) and sternotomy (Khalil et al., 2019).

Considering the experience of researcher regarding the anxiety of patients undergoing bone marrow biopsy and also since no study on the effect of lavender aroma on the anxiety of patients undergoing bone marrow biopsy has not been carried out in Iran, we decided to evaluate the effect of lavender aroma on anxiety of patients having undergoing bone marrow biopsy.

## Materials and Methods

*Search strategy and study selection*

The present study is a double blinded clinical trial with the code: N1 2016121431417, which was performed on 80 eligible patients who had been referred to Vali-e Asr hospital in 2017 for bone marrow biopsy.

To estimate the sample size, previous studies with the confident level of 95% and test power of 95% as well as consultation with a statistic’s exert were considered. The number of samples for each group was calculated to be 35.6 (36) using the following formula. However, this number was increased to 40 after considering the possibility of 10% sample drop. 

The samples were selected by convenience method using random blocks of 40, and then were equally divided into two groups of intervention (n=40) and control (n=40). Random sequence was generated by RAS software.

*Inclusion and exclusion criteria*

Inclusion criteria were; being 18 years old or older with confirmed diagnosis of leukemia or solid cell carcinoma, being alert to time, place and people at the time of data collection, having no hypersensitivity to lavender aroma, having the ability to smell, having minimal literacy, and having no history of mental illness. The exclusion criteria included; having severe cold, unwillingness to continue with the research, and patient’s death.

*Data extraction and quality assessment*

Data were collected using demographic questionnaire and Visual Anxiety Scale (VAS). The demographic questionnaire contained questions on age, gender, education level, marital status, occupation, disease diagnosis, biopsy (the first time or not), and physician experience. 

The VAS scale, which measures the level of anxiety, is a horizontal line that has been graded from 0 to 10. The researcher measured the anxiety level of samples based on the score they gave to their anxiety level, and classified them in the groups of without anxiety (0), with mild anxiety (1-3), with moderate anxiety (4-6), with severe anxiety (7-9) and with very severe anxiety (10).

The VAS questionnaire was designed by Lin et al to measure postoperative pain and anxiety (Lin et al., 2011). The validity of VAS questionnaire has been confirmed by Ottavian et al and Mohseni et al. Also, the reliability of VAS questionnaire was reported (r=0.83) by Rafiei et al in a pilot study with split-half method using Spearman Brown test. In the study of Zamanzadeh, the Cronbach’s alpha of the VAS questionnaire was found to be 0.83 (Zamanzadeh et al., 2015).

The researcher, after obtaining a written permission from the authorities and ethics committee of Tehran University of Medical Sciences, referred to Vali-e-Asr Hospital in Tehran, and after introducing herself as a researcher, explained the purpose of the study to patients and hospital managers. Then, written consent was obtained from the patients undergoing biopsy who had the inclusion criteria. Patients in the control group smelled 3 drops of distilled water on a cotton ball (which had been placed in a closed container) for 15 min at a distance of 7-10 cm, and their anxiety level was assessed immediately after the bone marrow biopsy using VAS scale. Patients in the intervention group smelled 3 drops of 10% lavender essential oil on a cotton ball (which had been placed in a closed container) for 15 min at a distance of 7-10 cm before the biopsy, and their anxiety level was assessed immediately after the bone marrow biopsy using VAS scale. Then, the results of both methods were compared.

It should be noted that, the amount of lavender aroma used in the intervention group was determine after consulting with and expert in traditional medicine. The researcher purchased the lavender essential oil from Barich Essence Company which is a valid company.

## Results

The results showed that 52.5% of samples in the control group were female and 55% of them in the intervention group were male. The mean age of samples in the control and intervention groups was 43±10.01 and 40±10.26, respectively. 

Most subjects in the control (90%) and intervention (85%) GROUPS WERE married. Also, 50% of samples in the control group and 45% in the intervention group were unemployed and in terms of education, 57% and 45% of samples in the control and intervention groups had high school diploma, respectively.

In terms of disease diagnosis, 60% and 45% of samples in the control and intervention groups had leukemia, respectively. Also, the highest percentage of subjects in both groups (80% in the control and 92.5% in the intervention groups) had history of other diseases. In terms of the history of biopsy, 62.5% and 80% of samples in the control and intervention groups had previous experience, respectively. Also, 40% of samples in both control and intervention groups were having biopsy from crest iliac site.

The results showed no significant difference (p> 0.05) between the two groups in terms of demographic characteristics, and they were relatively homogeneous (Table 1).

According to the results, the mean scores of anxiety in the control and intervention groups were 6.3 ± 1.92 and 3.75 ± 1.05, respectively. There was a significant difference (P <0.05) between the two groups in terms of anxiety score (Table 2). 

The results also revealed significant difference between the intervention and control groups in terms of the variables such as age, gender, physician experience, biopsy history and biopsy site (P <0.05). The anxiety score (-2.6) was lower in the intervention group than in the control group (Table 2).

Also, the results of regression analysis showed significant difference between the intervention and control groups (P <0.05) terms of the variables such as age, gender, physician experience, biopsy history and biopsy site (Table 3).

**Table 1:** Demographic Characteristics

Comparison		Control group Number (percentage)	Control group Number (percentage)	P-Value
Age (mean ± standard deviation)		43±10.01	40±10.26	0.396
Sex	Man	19 (47.5)	22 (55.0)	0.655
	Woman	21 (52.5)	18 (45.0)	
Marital status	Married	36 (90.0)	34 (85.0)	0.737
	Single	4 (10.0)	6 (15.0)	
Occupation	Unemployed	20 (50.0)	18 (45.0)	0.344
	Laborer	0	1 (2.5)	
	Office worker	10 (25.0)	13 (32.5)	
	Self-employed	7 (17.5)	8 (20)	
	Retired	3 (7.5)	0	
Education	Under diploma	9 (22.5)	9 (22.5)	0.408
	Diploma	23 (57.5)	18 (45)	
	University education	8 (20.0)	13 (32.5)	
Diagnosis	Leukemia	24 (60.0)	18 (45.0)	0.478
	Lymphoma	8 (20.0)	6 (15.0)	
	Osteosarcoma	2 (5.0)	6 (15.0)	
	Other	6 (15.0)	10 (25.0)	
Medical history (year)	0-5	32 (80.0)	37 (92.5)	0.193
	5-10	8 (20.0)	3 (7.5)	
History of bone marrow biopsy	None	27 (67.5)	32 (80.0)	0.360
	Second time	10 (25.0)	7 (17.5)	
	Several times	3 (7.5)	1 (2.5)	
Location of bone marrow biopsy	Iliac crest	40 (100)	40 (100)	0.057

**Table 2:** Mean Score of Anxiety Caused by Bone Marrow Biopsy in Control and Intervention Groups

Comparison	Control group (n = 40) Mean ± standard deviation	Intervention groups (n = 40) Mean ± standard deviation	Independent t-test		Analysis of covariance	
			MD (95% CI)	P-value	MD (95% CI)	P-value
Anxiety score	6/3 ±1/92	3/75± 1/05	-2/55 (-3/24-1/85)	P<0.001	-2/58 (-3/30-1/87)	P<0.001

Covariance analysis of confounding variables such as age, sex, physician experience, biopsy history and biopsy location

**Table 3:** Comparison of Anxiety Score in the Two Groups in Terms of the Confounding Variables based on Ranked Regression

Variable	Estimate	Lower limit of 95% confidence interval	Upper limit of 95% confidence interval	p-Value
Age	-0/52	-1/131	0.090	0.095
Gender	1/223	0/251	2/195	0.014
Physician experience	0/456-	-1/927	1/016	0/544
History of biopsy	-0/07	-0/968	0/827	0/878
Location of biopsy	-0/33	-1/955	1/295	0/691
Group Experimental	-3/374	-4/677	-2/081	0.0001
Control group	Ref	--	--	---

Ranked regression analysis of confounding variables such as age, sex, physician experience, biopsy history and biopsy location

## Discussion

The findings showed that patients in the control group experienced moderate to severe level of anxiety during bone marrow biopsy.

In the Brunetti study (2011), 73% of patients reported moderate to severe level of anxiety (4-10) during bone marrow biopsy (Brunetti et al., 2011). In the Shabanloei et al., (2010) study of anxiety during bone marrow biopsy, the results showed that patients in the control group reported an anxiety score of 52.18 ± 7.29. Lee et al., (2011) in a systematic study reviewed all studies that had investigated the effect of aromatherapy on anxiety level and found that, aromatherapy as a complication-free treatment had reduced the level of anxiety in almost all the studies. 

These results are in line with the findings of present study and show that bone marrow biopsy is associated with anxiety. 

In the Shahnazi et al., (2012) study, the anxiety level in the intervention and control groups during IUD insertion significantly reduced after smelling of lavender oil (p = 0.51). In the study of Abbaszadeh et al., (2018) with the aim of examining the effect of lavender aroma on the level of pain caused by bone marrow biopsy, the results showed that samples in the control group experienced a moderate to severe level of pain during biopsy and in fact, reducing the level of pain can in turn reduce the level of anxiety. 

In another study conducted by Khazaie et al., (2013)samples were randomly divided into three groups of lavender aroma dissolved in the sesame seed oil, sesame seed oil, and control. According to the results, the mean score of anxiety caused by IUD insertion decreased after intervention with lavender aroma (P <0.001). 

A study by Beyliklioğlu and Arslan, (2019) on patients undergoing breast surgery showed that smelling lavender oil had a significant effect on reducing anxiety of patients in the intervention group.

Safarabadi (2016) investigated the effect of aromatherapy with lavender on the anxiety of burn patients and showed a significant reduction in the anxiety of samples in the intervention group compared to the control group. 

The above studies are in line with the present study, suggesting that lavender aroma is effective in reducing anxiety.Contrary to the results of present study, in the study of Tayebi et al., (2015) aromatherapy with lavender essential oil only reduced the levels of depression and stress in hemodialysis patients and had no effect on their anxiety. This may be due to differences in the type of disease in these two studies. Although there is controversy about how aromatherapy reduces anxiety, the researchers believe that aromatherapy stimulates limbic system through olfactory nerves to release neurotransmitters such as noradrenaline, serotonin, endorphin and encephalin. In the study of Bagheri et al., (2017) which investigated the effect of lavender aroma on anxiety and depression of hemodialysis patients, the findings showed no significant difference between the two groups in terms of the severity of anxiety before the intervention and two and four weeks after the intervention. These results are not consistent with the findings of present study, although the researcher believes that dilution of lavender (5%) may have influenced the results of above study.

*Code of ethics*

IR.TUMS.FNM.REC.1395.780 

In conclusion, the results showed that lavender aroma was effective in reducing anxiety caused by bone marrow biopsy. Aromatherapy with lavender is an inexpensive, safe and non-invasive treatment that has many applications, so it is recommended to be used in the hematology and oncology clinics to reduce anxiety caused by bone marrow biopsy.

One of the limitations of this study was the previous experiences of patients undergoing bone marrow biopsy, which could affect the level of anxiety.

*Suggestion*

The present study investigated the effect of lavender aroma on anxiety of patients undergoing bone marrow biopsy. Some studies have suggested that massage with lavender oil can reduce fatigue, insomnia and pain in some patients (Bakhtshirin et al., 2015, Mohammed and Hassan, 2014). It is suggested to investigate the effect of massage with lavender oil on anxiety.
